# Temporal and spatial differences between taxonomic and trait biodiversity in a large marine ecosystem: Causes and consequences

**DOI:** 10.1371/journal.pone.0189731

**Published:** 2017-12-18

**Authors:** Tim Spaanheden Dencker, Laurene Pecuchet, Esther Beukhof, Katherine Richardson, Mark R. Payne, Martin Lindegren

**Affiliations:** 1 Centre for Ocean Life, National Institute of Aquatic Resources (DTU-Aqua), Technical University of Denmark, Kgs. Lyngby, Denmark; 2 Centre for Macroecology, Evolution and Climate, Danish Natural History Museum, University of Copenhagen, Copenhagen, Denmark; Sveriges lantbruksuniversitet, SWEDEN

## Abstract

Biodiversity is a multifaceted concept, yet most biodiversity studies have taken a taxonomic approach, implying that all species are equally important. However, species do not contribute equally to ecosystem processes and differ markedly in their responses to changing environments. This recognition has led to the exploration of other components of biodiversity, notably the diversity of ecologically important traits. Recent studies taking into account both taxonomic and trait diversity have revealed that the two biodiversity components may exhibit pronounced temporal and spatial differences. These apparent incongruences indicate that the two components may respond differently to environmental drivers and that changes in one component might not affect the other. Such incongruences may provide insight into the structuring of communities through community assembly processes, and the resilience of ecosystems to change. Here we examine temporal and spatial patterns and drivers of multiple marine biodiversity indicators using the North Sea fish community as a case study. Based on long-term spatially resolved survey data on fish species occurrences and biomasses from 1983 to 2014 and an extensive trait dataset we: (i) investigate temporal and spatial incongruences between taxonomy and trait-based indicators of both richness and evenness; (ii) examine the underlying environmental drivers and, (iii) interpret the results in the context of assembly rules acting on community composition. Our study shows that taxonomy and trait-based biodiversity indicators differ in time and space and that these differences are correlated to natural and anthropogenic drivers, notably temperature, depth and substrate richness. Our findings show that trait-based biodiversity indicators add information regarding community composition and ecosystem structure compared to and in conjunction with taxonomy-based indicators. These results emphasize the importance of examining and monitoring multiple indicators of biodiversity in ecological studies as well as for conservation and ecosystem-based management purposes.

## Introduction

Understanding patterns of biodiversity and their underlying drivers has challenged scientists for centuries [1,2], and it remains a fundamental and strongly debated field in ecology [3]. Biodiversity is a multifaceted concept comprising several components, as recognized by the Convention of Biological Diversity [4], and yet biodiversity studies have traditionally focused on taxonomic units to describe patterns and drivers of biodiversity (species richness and abundance distribution) at various spatial scales [2,5,6]. These biodiversity indicators include no other information than the taxonomic identity of the species and imply that all species are equally important. However, it is well known that species differ in their contribution to ecosystem processes [7], and that they exhibit marked differences in their responses to changing environments. This recognition has led to the exploration of components of biodiversity other than taxonomic diversity in ecosystems and species assemblages.

One such component is the diversity of ecologically important traits, often referred to as “functional diversity” [8,9]. Traits are defined as measurable attributes affecting the fitness of organisms through the processes of feeding, reproduction and survival [10,11]. These attributes can be morphological (e.g. size and body shape), physiological (e.g. metabolic pathways or growth related) or behavioral (e.g. diurnal migration, feeding patterns). Together, combinations of traits can describe the ecological niche of species [12,13]. Furthermore, traits determine the response of species to environmental gradients and perturbations [14] and provide insight into the functional role of species in ecosystems [15]. Recently, terrestrial and marine studies taking into account multiple components of biodiversity using both taxonomic and trait information have revealed that the two components of biodiversity may exhibit temporal and spatial differences [16–19]. These apparent discrepancies indicate that the two components of biodiversity may respond differently to environmental drivers and perturbations [20,21].

Furthermore, these differences between species and trait diversity can provide insight into the key mechanisms and processes structuring biological communities [22,23]. Local communities may display greater, or lesser, trait diversity than expected from of a random selection of species from a regional species pool. The resulting patterns of so-called over- or underdispersion of traits may be indicative of the effects of abiotic or biotic forces acting on community assembly, through the processes of environmental filtering or limiting similarity, respectively [24]. Environmental filtering is hypothesized to lead to trait homogenization in communities as only species with a specific set of traits might survive and thrive under certain abiotic conditions. Limiting similarity, on the other hand, acts mainly through biotic processes, as competition over limiting resources leads to separation of niches and increased trait heterogeneity [25].

In addition to the structuring mechanisms of environmental filtering and limiting similarity marine fish communities have been and are heavily altered by fishing at global and regional scales [26–28]. The composition of fish communities might be affected by changes in the biomass of targeted and bycatch species and especially by the strong structuring effect of size-selective harvesting (e.g. trawling), which typically targets large individuals, thereby reducing trait variability and shifting the abundance distribution of the community towards smaller individuals, while not necessarily affecting the number of species, i.e. species richness [29].

The potential resilience of ecosystems to such anthropogenic and natural stressors may also depend on the ratios between different components of biodiversity [30]. The loss of species with unique functional traits may have more severe consequences on ecosystem functioning compared to the loss of species with traits that are more commonly expressed within the community [31]. This redundancy is however highly variable across ecosystems. For instance, certain Argentinean plant communities could lose 75% of their species before any unique functional group would disappear [32], while some coastal fish and avian assemblages exhibit low degrees of functional redundancy, thus revealing high vulnerability to species loss [30,33,34].

**Fig 1:**
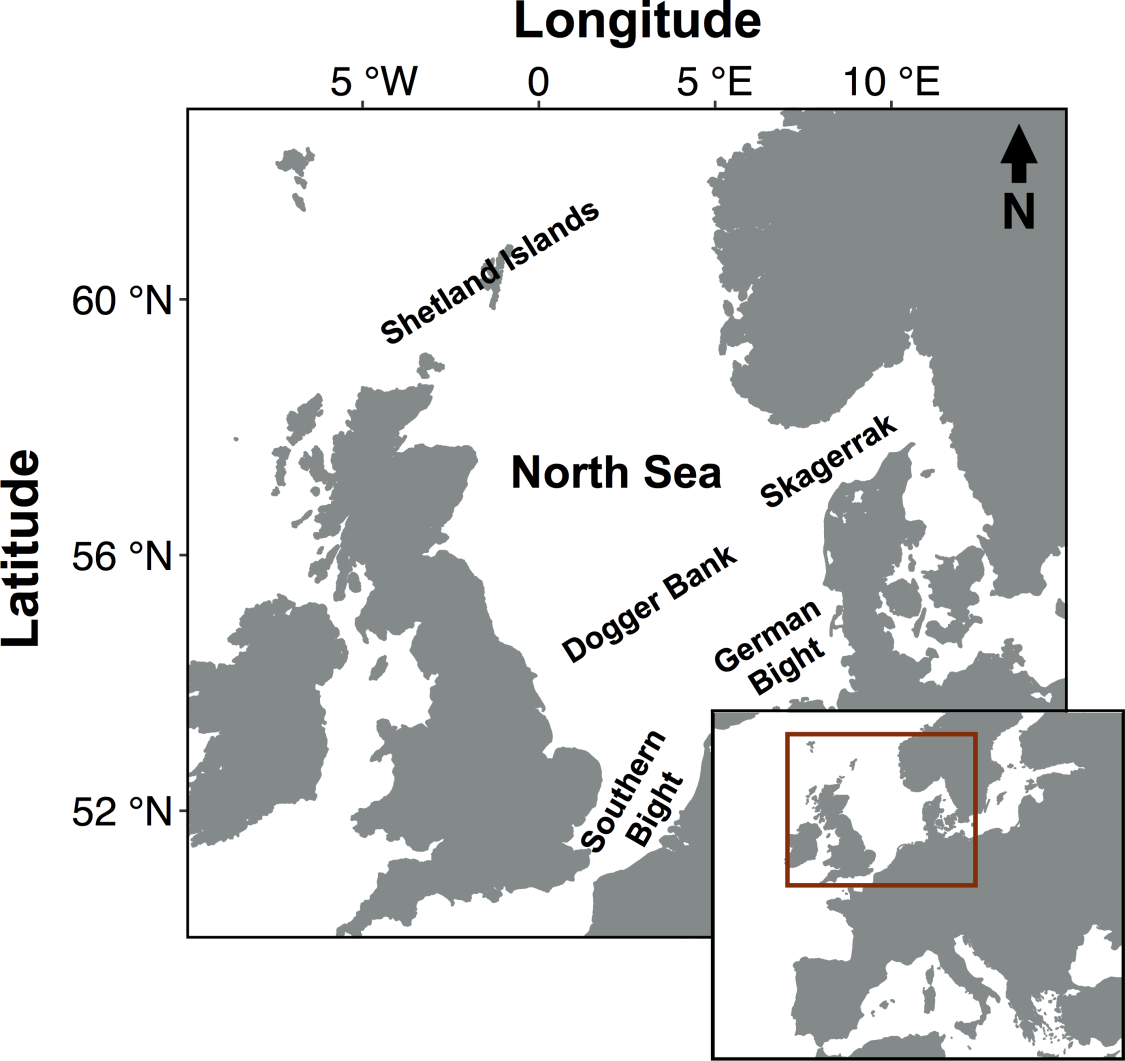
Map of the North Sea and its geographical position. Labels correspond to the names of specific localities in terms of areas and geographic features including banks, bights and islands mentioned in the study.

Disentangling and decoupling the temporal and spatial dynamics of species diversity and trait diversity is therefore critical for elucidating the drivers and processes of community assembly [23,35], and for developing an understanding of the effect of biodiversity loss on ecosystem functioning [36]. In addition, such an understanding can provide valuable input for informing and planning broad-scale conservation and ecosystem-based management strategies. Here, we examine spatial and temporal patterns and compare drivers of multiple marine biodiversity indicators using the North Sea demersal fish community as a case study. The North Sea (Fig 1) is a heavily impacted large marine ecosystem [27] that has experienced rapid changes in environmental conditions [37] and shifting community compositions [37,38]. Using an extensive trait dataset and standardized long-term spatially resolved survey data on fish species occurrences and abundances, we: (i) investigate the temporal and spatial differences between taxonomy and trait-based biodiversity indicators, (ii) assess the importance of environmental drivers on the observed biodiversity patterns, and (iii) interpret the results in the context of assembly rules acting on community composition and ecosystem resilience.

## Materials & methods

### Fish survey data

Distribution and abundance data for demersal fish species were obtained from the North Sea International Bottom Trawl Survey (NS-IBTS), publicly available from the ICES trawl surveys data base [39]. As survey methods have been standardized among all participating countries since 1983, data on Catch per Unit Effort (CPUE; catch in numbers of individuals of the same species adjusted to one hour of trawling) per length class were extracted from 1983 to 2014 for the months of January to March (hereafter referred to as *quarter* one). To avoid potential bias related to changes in the sampled survey area over time, only ICES statistical rectangles (1° longitude × 0.5° latitude; hereafter *ICES rectangle*) that were sampled in at least 26 out of 32 years (80%) were used in the analysis. In order to standardize haul duration, only hauls with duration lengths of between 27 and 33 minutes (median haul duration of 30 minutes ± 10%) were retained. All invertebrate and pelagic fish species were removed from the dataset, limiting the analysis to demersal fish species. In addition, a minimum hauling depth of 20 meters was selected to exclude samples which might represent coastal or estuarine areas, as these areas are not prioritized in the survey. To minimize the effect of misidentifications or sporadically occurring species due to the effects of inadequate sampling, only species that were present in at least 7 out of 32 years (20%) were kept for further analyses. This selection criterion excluded 27 species. We acknowledge that the criterion might have an effect on the number of rare species reported but not on the species that show consistent recurrence or increase over time. Furthermore, a few ecologically similar species of the same genus were aggregated due to identification problems in the reporting scheme [40] and the lack of trait information (S1 Table). For consistency, we refer to all species and species aggregates as species. Using length-weight parameters for each species, CPUEs per length classes were converted into biomass caught per hour following [41]. Conversion parameters and relative biomass of species are outlined in S2 Table and S1 Fig Species biomasses per year per ICES rectangle are reported in S1 Dataset. The data corrections resulted in a dataset containing 9401 unique hauls in 119 ICES rectangles and biomass catch per hour for 77 demersal fish species.

### Fish trait data

Eight ecological trait categories were used to summarize community biodiversity. The selected trait categories are related to the morphological, life history, reproductive or dietary aspects of marine fish species, and have been shown to determine structure and function in marine fish communities (Table 1). Morphology of the fish species was described using body size, body shape and caudal fin shape. Life history was covered by age at maturity, while reproductive and dietary aspects were captured by, respectively, offspring size, fecundity and spawning behavior, and diet. The set of traits was selected to reflect different and complementary aspects of the ecological niche of the species, and this trait set has a high degree of resemblance to sets used in similar multi-trait studies [15,17,23,42]. Trait information was extracted from the primary literature and Fishbase [43] (S3 Table). Since trait data were not available from the North Sea for all the species, some trait data were also derived from neighboring areas (such as the Baltic Sea) or from the larger North Atlantic regions.

**Table 1:** Overview of the eight selected trait categories sorted according to traits, description and ecological relevance.

Trait	Trait categories	Description	Relevance
Body size	Continuous	Length a fish would reach if it was to grow indefinitely	Information on food web structure and ecological niche occupation
Age at maturity	Continuous	Age at which 50% of the individuals are sexually mature	Relates to lifespan and generation time
Fecundity	Continuous	Average number of eggs per adult female during a spawning season	Relates to energy output, allocation and production
Egg size	Continuous	Size of oocyte at spawning	Relates to spawning behavior and offspring investment
Body shape	Gadoid-like	The shape of the	Insights into predation
	Flat	body	behavior, mobility and
	Elongated		habitat selection
	Short/deep		
	Eel-like		
Diet	Benthivore	Main dietary	Insights into the trophic
	Piscivore	group(s)	structure of
	Planktivore		communities
	Bentho-piscivore		
	Plankto-piscivore		
Spawning behavior	Ob—Oviparous with benthic	Main spawning	Relates to ecological
	eggs	behavior, divided	constraint on habitat
	Og–Oviparous guarders	between oviparity	selection [44]
	Op—Oviparous with pelagic	and vivparity, and	
	eggs	further between the	
	Os–Oviparous shelterers	degree of parental	
	Ov—Oviparous with adhesive eggs	care, mode of release and egg	
	V—Viviparous	characteristics	
Caudal fin shape	Truncated	The shape of the	Relates to habitat
	Continuous	caudal fin	selection and activity
	Forked		
	Rounded		
	Emarginate		
	Heterocercal		

### Biodiversity indicators

Four commonly used indicators of biodiversity were calculated: species richness (SRic), species evenness (SEve), trait richness (TRic) and trait evenness (TEve). SRic was calculated as the number of unique species, while SEve was calculated as Pielou’s evenness [45]. The value of Pielou’s evenness ranges from 0 to 1, with larger values indicating a more even distribution in relative biomass among species in a sample. The trait-based biodiversity indices follow the proposed mathematical formulas suggested by [46,47], allowing for standardizing of trait values, and are calculated based on all eight traits. Both TRic and TEve are represented by a multidimensional trait space. TRic represents the multidimensional trait space occupied by the community calculated as the minimum convex hull volume which includes the trait values of all species considered [47]. TRic was standardized between 0 and 1, with larger values indicating a larger convex hull volume, hence a higher richness of traits in a sample. TEve was defined as the evenness of the distribution of relative biomass of species in the trait space [9], and ranges, as in the case of SEve, from 0 to 1, depending on the degree of evenness in the distribution of biomass among traits in a sample. TRic and TEve were chosen to be comparable to their taxonomy-based equivalents, respectively SRic and SEve. The taxonomy and trait-based indicators were calculated following standard approaches implemented in the R packages “vegan” [48] and “FD” [46]. All biodiversity indicators were calculated per ICES rectangle per year and then averaged across either ICES rectangles or years to investigate temporal trends and spatial patterns, respectively. Temporal trends were assessed with generalized additive models (GAMs) [49] with a smoother function of *year* as the single predictor. No temporal autocorrelation was detected in the residuals. As the number of hauls conducted in each ICES rectangle per year varied from 1 to 11 (mean: 2.0, median: 2.9), all biodiversity indicators were standardized for differences in sampling size by using GAMs which effectively accounts for potential non-linear relationships. Values for each biodiversity indicator per year per ICES rectangle are reported in S2 Dataset

### Natural and anthropogenic environmental drivers of biodiversity

To investigate potential drivers of species and trait diversity, ten natural and anthropogenic environmental drivers were selected as covariates. The drivers were selected based on their demonstrated importance in shaping patterns of fish biodiversity in marine ecosystems [2,23,50]. Only spatial patterns of biodiversity were investigated due to two reasons: the highest variability was found across spatial scales, and not all drivers were fully available across the full temporal scale of the study. Depth was calculated by averaging the depth of sampled hauls per ICES rectangle from the NS-IBTS data. Sea bottom temperature (°C) and sea bottom salinity data were obtained from Núñez-Riboni & Akimova (2015) [51] on a monthly basis with a resolution of 0.2° × 0.2°. Mean winter (Dec-Feb) sea bottom temperature and salinity were derived per ICES rectangle per year. Temperature seasonality was expressed as the difference between winter and summer (Jun-Aug) temperatures for each ICES rectangle. Salinity variability was expressed as the difference between minimum and maximum salinity within each ICES rectangle per year and then averaged across years. Phytoplankton biomass was estimated by proxy using the Phytoplankton Colour Index (PCI) [52] during quarter one and retrieved from the Continuous Plankton Recorder program provided by the Sir Alister Hardy Foundation for Ocean Science [53]. PCI is a semi-quantitative index that provides an estimate of phytoplankton biomass based on the greenness of water samples [54]. PCI data were available for the entire study period, but not for the whole study area in every year, hence spatial interpolation of this data source was performed using a GAM with a two-dimensional (latitude, longitude) tensor product smoother. Phytoplankton biomass was represented by mean quarter one PCI per ICES rectangle across all years. Seabed substrate richness and evenness were calculated based on seabed substrate classifications from The European Marine Observation and Data Network [55]. Six different substrate categories were used and substrate richness was defined as the number of categories present in each ICES rectangle. Substrate evenness was calculated as Pielou’s evenness, based on the relative coverage of substrate categories within each ICES rectangle. Anthropogenic pressure from fishing was estimated from data on the spatial distribution of international bottom trawling effort in the North Sea for two separate periods: 1990–1995 [56] and 2003–2012 [57,58]. Beam and otter trawl effort were considered separately as recommended by Engelhard *et al*. [58]. Data, summary statistics and sources of environmental covariates can be found in the supplementary material (S3 Dataset and S4 Table).

### Modelling

To investigate the relative importance of natural and anthropogenic drivers in explaining the spatial patterns of biodiversity, we fitted a series of GAMs to each indicator of biodiversity. GAMs are non-parametric modelling methods that allow a high degree of flexibility in the form of the response [49]. The relationship between biodiversity indicators and drivers was only investigated for spatial patterns, as complete temporal coverage was not available for the entire study period. Two sets of GAMs were performed: one using the mean values of all natural drivers over the entire study period; and one using a reduced data set containing mean values of all natural *and* anthropogenic drivers for the two periods in which fishing effort data were available. All GAMs were performed with a Gaussian error term and restricted to a three degrees of freedom smoother (k = 3), equivalent to a second degree polynomial. Instead of a traditional model reduction procedure, each covariate was considered for inclusion and could reasonably be considered as having an effect, despite failing to meet an *a priori* determined significance level of p<0.05 [59–61]. Instead, the importance of each covariate was assessed using relative variable importance (RVI) from the R package ‘MuMIn’ [62] based on weighted Akaike’s Information Criterion (AIC) [63]. The higher the RVI for an explanatory variable, the more important it is for explaining the spatial patterns of the biodiversity indicators [60]. No spatial autocorrelation was detected in the residuals of the spatial GAMs.

### Null model—detecting assembly processes

To investigate potential assembly processes impacting the community composition we compared observed spatial values of TRic with simulated TRic values obtained from a null model, based on 999 randomized species assemblages taken from the observed species pool. Randomizations were obtained by controlling for both row sums (sites; i.e. ICES rectangles) and column sums (species) using the ‘permatswap()’ function in the ‘vegan’ package in R [48]. If assemblages have higher TRic than expected from a null model at a given level of SRic, these assemblages will be influenced mainly by limiting similarity, while assemblages with lower TRic than expected from a null model will be influenced mainly by environmental filtering. The deviance of the observed TRic from the simulated TRic was considered as an indication of the relative importance of the two suggested assembly rules. Values within the interquartile range corresponded to assemblages where neither of the assembly rules dominate, while values below or above the 25% and 75% quartiles, respectively, indicate assemblages predominantly structured through either environmental filtering or limiting similarity. Assemblages with values outside the 95% range were considered to be significantly different from the null-model and to be strongly structured through either environmental filtering or limiting similarity. All statistical analyses were performed in R (version 3.3.2) [64].

## Results

### Tempo-spatial patterns of biodiversity

**Fig 2:**
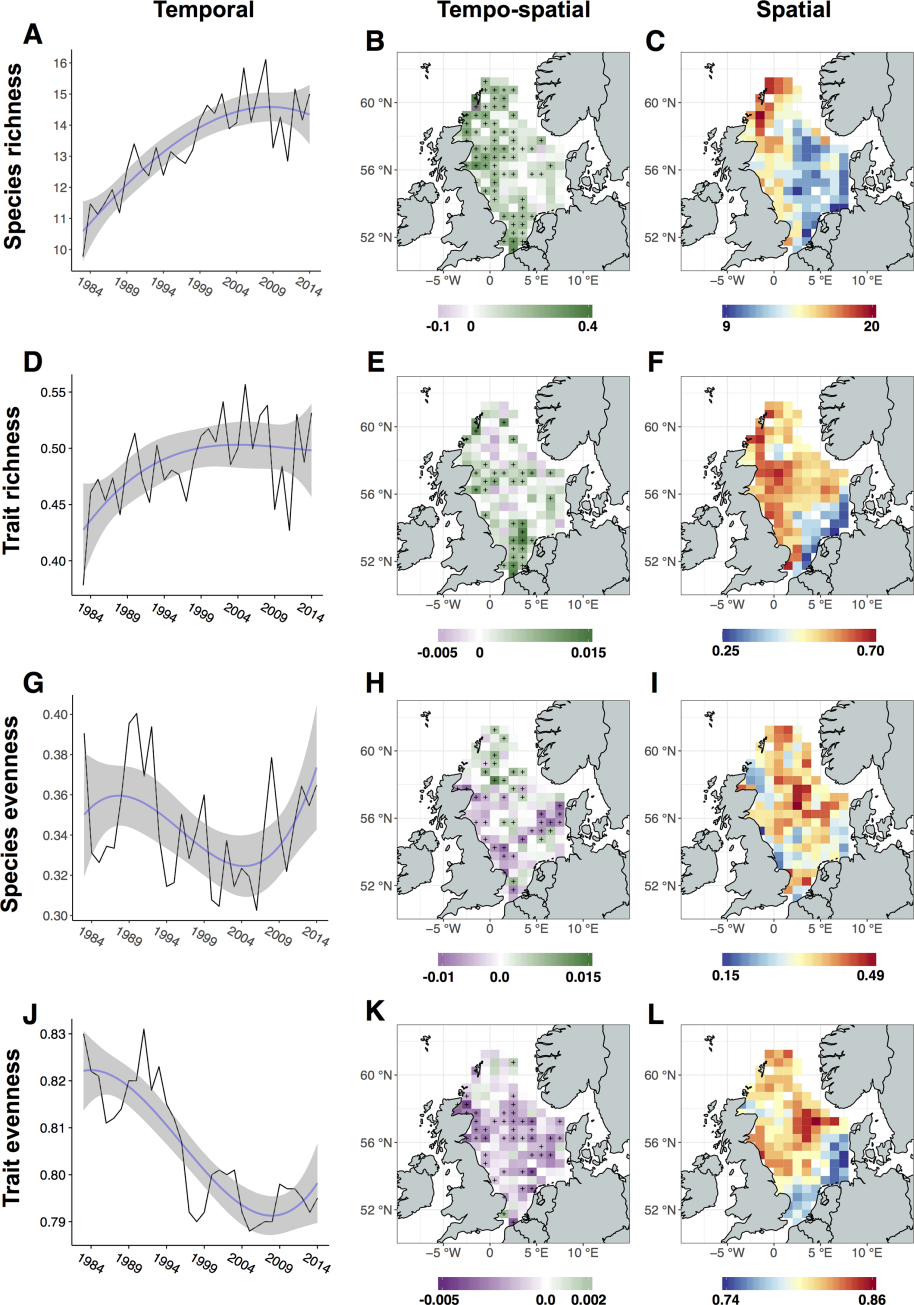
Temporal trends and spatial patterns of multiple biodiversity indicators in the North Sea. (A, D, G, J) Time-series and temporal trends of fish species richness (SRic), trait richness (TRic), species evenness (SEve), and trait evenness (TEve) as annual averages across all ICES rectangles. Significant temporal trends were observed for SRic (GAM: F = 37.45, e.d.f. = 1.92, R^2^ = 0.64, p < 0.001), TRic (GAM: F = 5.6, e.d.f. = 1.78, R^2^ = 0.33, p < 0.01) and TEve (GAM: F = 39.84, e.d.f. = 1.75, R^2^ = 0.71, p < 0.001). Shaded grey represent 95% confidence intervals. (B, E, H, K) Tempo-spatial patterns of biodiversity indicators represented by the slope and significance of a linear regression model fitted to each ICES rectangle across years. Green colors indicate a positive trend, while purple colors indicate a negative trend. Significant trends are indicated by black crosses (p<0.05). (C, F, I, L) Spatial patterns of biodiversity indicators shown as average value for each ICES rectangle across all years.

The average SRic per ICES rectangle showed a significant long-term increase with a recent stagnation from 2005 onwards (Fig 2A). This trend is reflected also in TRic, albeit with a more moderate increase (Fig 2D). TRic was significantly positively correlated to SRic (GAM: F = 92.28, e.d.f. = 1, *R*^*2*^ = 0.75, p < 0.05), although a significant decrease in the ratio between TRic and SRic was observed during the study period (GAM: F = 87.3, e.d.f. = 1.85 *R*^*2*^ = 0.75, p < 0.05), indicating that TRic did not increase at the same rate as SRic (S2 Fig). The increase in SRic was reflected by a significantly increasing trend in 51% of the ICES rectangles (Fig 2B), mainly found in rectangles located in the northwestern and southwestern North Sea. TRic increased primarily in the southwestern and western central North Sea, coinciding with areas of increases in SRic. However, only 26% of the ICES rectangles showed a significant increase in TRic, indicating a more localized extent of increase compared to SRic (Fig 2E).

With respect to the evenness indicators, SEve showed pronounced fluctuations, but no significant temporal trend was detected throughout the study period (Fig 2G), while TEve showed a significant long-term decrease (Fig 2J). SEve was generally characterized by low values, ranging from 0.3 to 0.4, in contrast to TEve, where observed values ranged from 0.78 to 0.83. SEve decreased significantly in 16% of the ICES rectangles, primarily in the southern North Sea, with a distinct band across the central North Sea following the northern border of Dogger Bank. The northern North Sea was characterized by significant increases in SEve, although over a more restricted area than the observed decreases in the southern North Sea (Fig 2H). TEve decreased significantly in the southeastern, central and western regions of the North Sea, whereas little temporal change was detected in the northern parts (Fig 2K).

In terms of spatial patterns, the highest values of SRic were observed in the northern North Sea from the coast of Scotland to the Shetland Islands, whereas the areas with lowest SRic were found in the central and southeastern North Sea (Fig 2C). Areas with medium to high values of SRic were observed along the British coast, coinciding with the highest values of TRic, and in the eastern parts along the Danish coast (Fig 1F). TRic was observed to be consistently high along the British coast with intermediate-values in the northern and central-eastern North Sea. Low values of TRic were found in the southeastern North Sea, with the lowest values in coastal areas. Several transition zones were identified, marking steep changes in biodiversity values between adjacent areas. SRic was observed to decrease markedly towards the central and southeastern North Sea, while two distinct transition zones were found for TRic. A first transition zone was found at Dogger Bank with high values to the west and north, while low values were observed south and east of the bank. Secondly, the Southern Bight was clearly split between a western and eastern component with high values of TRic on the British coast and low values on the Dutch and Belgian coast. SRic and TRic show a high degree of overlap with two exceptions: the area with maximum values of TRic is situated farther south than the area for SRic. Moreover, the northern central North Sea is characterized by low SRic, but by mid to high levels of TRic. No strong spatial pattern was observed in the average values of SEve, though it showed a marked peak in values in the central North Sea (Fig 2I). The spatial pattern of TEve, on the other hand, was marked by a clear transition over Dogger Bank, with lower values in the southern and southeastern part and higher values towards the British coast and into the central and northern North Sea (Fig 2L).

### Drivers of biodiversity

**Fig 3:**
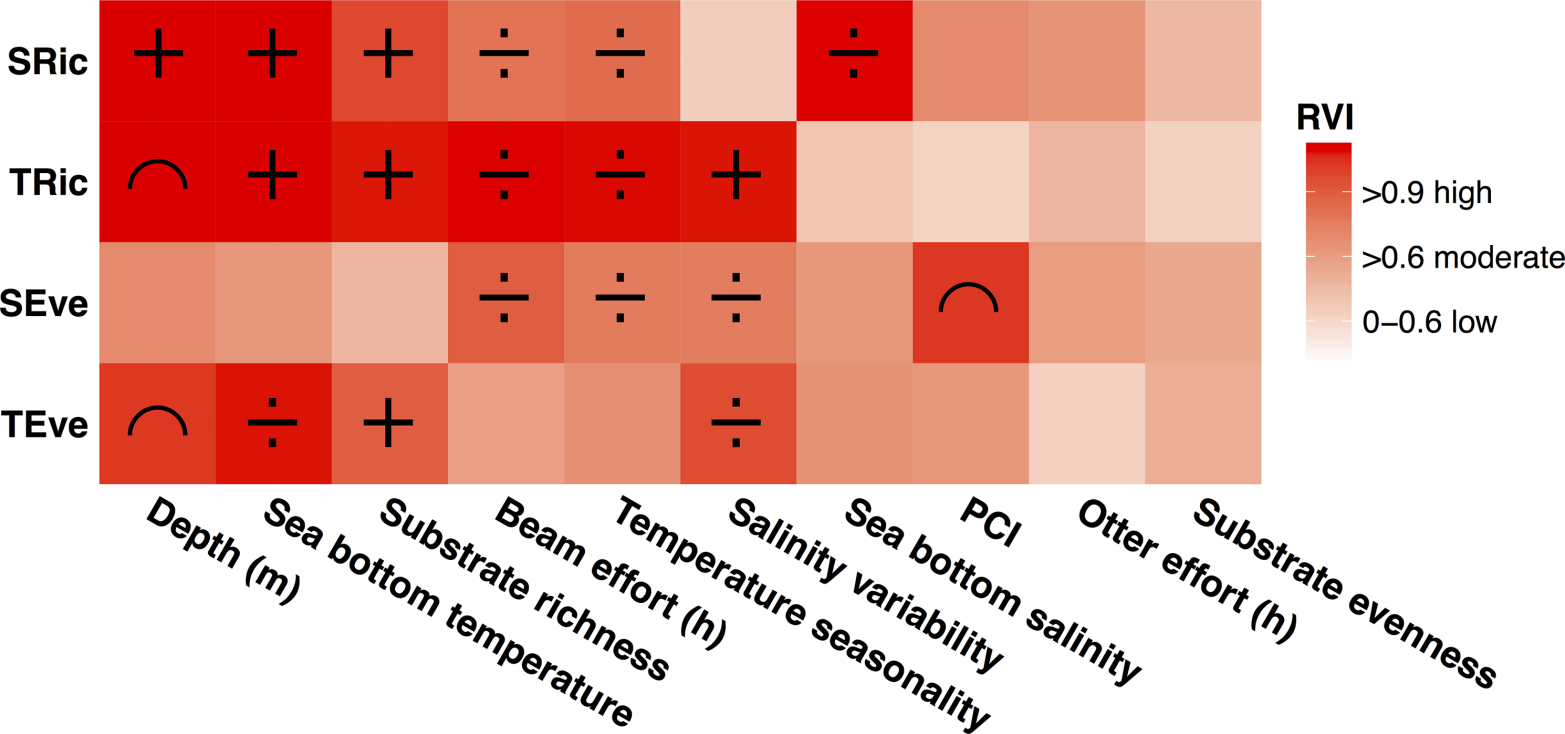
Relative variable importance (RVI) of environmental and anthropogenic drivers and their relationship to the biodiversity indicators. Drivers are sorted according to their cumulative importance across the four investigated biodiversity indicators. RVI>0.9 signifies high importance of a driver, RVI>0.6 signifies moderate importance, while RVI<0.6 is considered low or no importance. Relationships between drivers and biodiversity indicators based on GAMs are indicated by symbols: **+** indicate a positive relationship, **÷** indicate a negative relationship, **∩** indicate a unimodal relationship. If no symbol is assigned, the RVI of the driver is below 0.6.

The spatial GAMs explained 76% and 36% of the spatial variability of SRic and SEve, while 55% and 69% of the spatial variability was explained for TRic and TEve, respectively. The most influential drivers across the four diversity indicators were depth, sea bottom temperature and substrate richness, followed by beam trawl effort, temperature seasonality and salinity variability. The relative importance of the drivers varied, however, between biodiversity indicators (Fig 3). Drivers of SRic and TRic showed a high degree of agreement with respect to the importance of drivers, and their relationship to the biodiversity indicators. Both richness indicators were positively related to sea bottom temperature and substrate richness, and negatively related to beam trawl effort and temperature seasonality. For depth, SRic was observed to follow a positive relationship, while TRic peaked at intermediate depths of around 80–100 meters. A low degree of congruence was observed between the importance of drivers on the evenness indicators. Only salinity variability was found to be important for these and a negative relationship was found for evenness indicators. Additionally, beam effort, temperature seasonality and PCI were important for SEve, where a negative relationship was found for the first two drivers, and a unimodal relation for PCI. Depth, sea bottom temperature, and substrate richness were the most important drivers for TEve, in addition to salinity variability, with unimodal, negative and positive relationships, respectively.

### Observed TRic against null model

**Fig 4:**
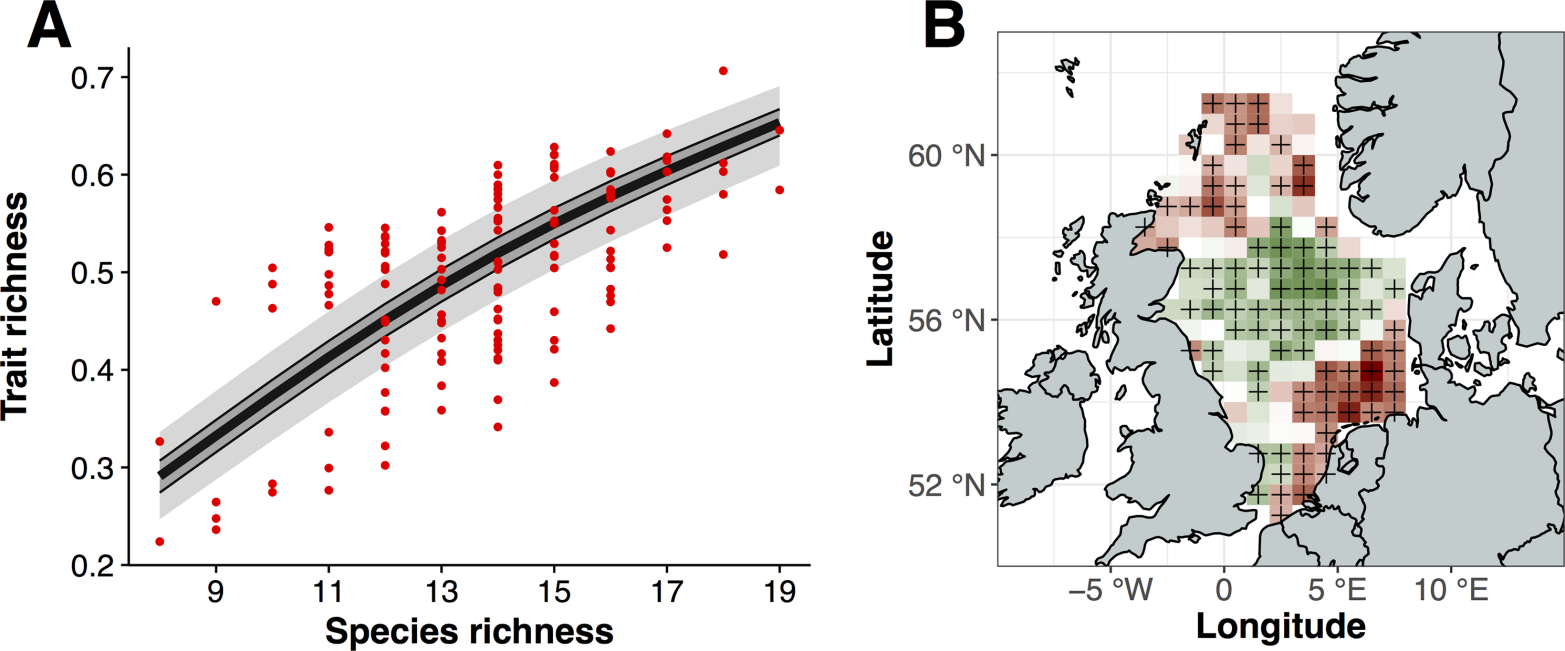
Null model results and the spatial distribution of over- and underdispersion in the North Sea. (A) Observed (red dots) and simulated trait richness (TRic) values based on a null model. Bold black line: mean of 999 random permutations; areas shaded in dark and light grey: 50^th^ and 95^th^ percentiles, respectively, smoothed using a generalized additive model (GAM) function. (B) Spatial distribution of residuals of observed TRic from the null model. Areas shaded in green and red are characterized by over- and underdispersion, respectively, where the observed TRic is outside the 50^th^ percentiles is either higher or lower than expected from its level of SRic. Black crosses (+) indicate significant deviation from the null model as described by falling outside the 95^th^ percentile of simulated values.

TRic and SRic showed a positive relationship, which is reflected also in the simulated null-model relationship (Fig 4A). For low levels of SRic (<11 species), the observed TRic values were primarily distributed outside the 95% range, thus being significantly different from the null-model distribution. For higher levels of SRic (≥11 species), the observed TRic occupied both areas outside and inside the 95% interval. The spatial distribution of residuals of observed TRic from the null-model was characterized by a clear northern and southern component of significantly lower values than expected from the null model, notably in the German Bight and in the northern North Sea between the Shetland Islands and Norway. Areas characterized by higher than expected TRic were observed primarily in the central North Sea following a west-to-east band cutting across from the British coast to Skagerrak (Fig 4B)

## Discussion

Our study documents pronounced differences in temporal trends and spatial patterns between multiple components of demersal fish biodiversity in the North Sea, including taxonomic identity and ecological traits. Below we elaborate on these incongruences, discuss their underlying causes and drivers, and discuss the associated ecological consequences.

### Differences in time and space

Despite similar increasing temporal trends, the close match between SRic and TRic starts to break down when the spatial dimension is taken into account. While similar increases in both SRic and TRic were observed throughout the Southern Bight, a limited degree of spatial overlap was found in the northern North Sea. This suggests that species gains in the southern Bight have contributed with novel trait values, while the widespread increase in SRic noted in the northern North Sea has contributed only locally to novel trait values. The observed spatial differences can be driven by the introduction of new species and range shifts of already existing species. Interestingly, the distribution range of species with different biogeographic affinities has shifted unevenly within the North Sea [65] with small-sized Lusitanian species expanding their distribution ranges compared to large-bodied Boreal (northerly) species [37]. Thus, their expansion into the North Sea can likely explain the contribution of new trait values, particularly along the “entry point” in the southern North Sea, i.e. the Southern Bight. The high degree of spatial differentiation in the contribution of new species and traits into existing local assemblages highlights that immigration from adjacent regions into the North Sea is an important factor in structuring fish diversity and community composition.

As in the case of the temporal dynamics described above, the spatial patterns of the biodiversity indicators displayed both similarities and differences. The similarities are illustrated by a pronounced north-south gradient in both SRic and TRic. This supports earlier studies showing a clear separation in community composition between the northern and southern North Sea [38,66,67]. Despite these similarities, the two indicators demonstrate pronounced local differences, particularly in the central North Sea—an area characterized by considerably higher TRic compared to its corresponding level of SRic. This indicates a high degree of trait heterogeneity between assemblages across levels of species richness. This is in accordance with findings from the Barents and Baltic Seas demonstrating similar spatial differentiation between species richness and trait richness, albeit at lower levels of species richness [23,42].

It is recognized that the ecological effect of a species on ecosystem processes is generally proportional to its relative biomass in the community [68], with the notable exception of keystone species showing a disproportionally large effect compared to their biomass [69,70]. Furthermore, biodiversity patterns depend not only on the presence and absence of the species and their traits, but also on their relative abundance and biomass [17]. In order to account for species biomasses, we therefore included indicators of species and trait evenness. The North Sea was generally characterized by low SEve during the study period which suggests a community with a few dominating species (e.g. whiting (*Merlangius merlangus*), common dab (*Limanda limanda*), and Atlantic cod (*Gadus morhua*)). This is in contrast to TEve which despite showing a significant decrease over the study period, remained relatively high over time and throughout space. These contrasts may indicate a community characterized by a few dominant species, but also with high regularity in the distribution of biomass across traits. Similar differences in evenness indicators have previously been found for tropical fish across several lagoon systems [21]. Many marine ecosystems have been impacted by marked environmental changes [27] and the North Sea fish community has undergone major distributional shifts during the last four decades especially due to increasing sea bottom temperatures [71]. These shifts, in addition to the effect of fishing and the appearance of novel species potentially affected the relative biomass distribution across species and traits. Shifts in evenness patterns can lead to changes in interspecific interactions, ecosystem processes and ecosystem stability [72]. More importantly, evenness indicators might respond more rapidly to changes in communities than species or trait richness, as changes in abundances or biomass often precede local species extinctions [73].

### Natural and anthropogenic drivers

The observed similarities and differences between biodiversity indicators suggest that the investigated components of the North Sea demersal fish biodiversity may respond differently to environmental and anthropogenic drivers. One of the most influential drivers in this study, sea bottom temperature had a positive effect on both SRic and TRic. This suggests that temperature is an important driver for structuring of communities by determining patterns of species occurrences. This is in concordance with previous studies linking temperature increases to changes in community composition [2,74–76]. In addition to temperature, depth was found one of the most influential explanatory variables. Although depth has shown to be a suitable predictor for community structure [50,67,75] it is unlikely the actual driving force behind the observed patterns, but rather a proxy for factors of more direct influence, such as water column mixing or geographical proximity to the highly diverse species pool of the Northeast Atlantic. In addition to temperature and depth, substrate richness demonstrated a strong positive relationship with all biodiversity indicators, except SEve. This supports the *habitat heterogeneity hypothesis* [77], stating that structurally more complex habitats provide more niches thereby increasing species and trait richness. The higher evenness, particularly in terms of traits may also be linked to the higher number of niches available in environments with high habitat heterogeneity. More niches may support a more diverse community at relatively even abundances compared to niche poor environments, where single or few species might dominate. This opposite end of the spectrum may be evident in the southeastern North Sea, which was characterized by both low species and trait richness, as well as by low species evenness and low substrate richness.

In addition to the natural drivers, three out of the four biodiversity indicators were negatively correlated to beam trawl effort, but uncorrelated to otter trawl effort. Although fishing can significantly impact marine communities [78], particularly in terms of evenness (i.e. by affecting the underlying population abundances of target and non-target species), the negative correlation may not necessarily reflect a true effect on the biodiversity indicators, but rather may be a result of the clear spatial preference of the beam trawl fisheries for the southern North Sea. This preference has previously been explained by external environmental factors such as primary productivity, depth and sediment grain size, largely favoring the main targeted flatfish species [79]. However, the potential effects of trawling have been investigated in other studies [58] and historical records show that the southern North Sea used to have a much higher proportion of large-bodied elasmobranchs and diadromous fishes [80,81] than is the case today. This suggests that fishing has had a clear effect on community composition in the southern North Sea. In addition, fishing pressure affects fish communities non-randomly, often targeting large, predatory species and individuals [29,82,83], leading to changes in both the presence and abundance of certain key traits, such as body length, which may lead to a loss of trait heterogeneity and potentially affecting the trophic structures of marine communities [84]. The historical records and the non-random effects of fishing pressure highlight the need to adopt trait-based approaches in long-term perspectives to understand fishing impacts on community composition and marine ecosystems. One such example is the Large Fish Indicator [85], indicating the proportion of large fish (>40 cm) in the North Sea demersal fish community. The indicator has been used to detect the positive effects of recent effort reductions in the North Sea fishing fleet [58].

### Causes and consequences of differences between biodiversity indicators

Assessing differences between biodiversity components can provide information on the underlying abiotic or biotic processes shaping community assembly [22]. The null model results revealed areas where local assemblages are either more or less diverse in traits than if assembled at random and illustrated a clear spatial separation between assembly processes in the North Sea. The significant underdispersion of the southeastern North Sea indicate a strong effect of environmental filtering acting on community composition through a stressful habitat characterized by pronounced seasonal fluctuations in temperature and salinity, low substrate richness and shallow depths. These environmental conditions, along with pronounced bed stress via waves and tides, as well as bottom trawling, make the southern North Sea a relatively stressful environment, where only species with a limited set of traits enabling them to cope with these conditions can exist. In contrast, the central North Sea is characterized by pronounced overdispersion, wherein biotic interaction and resource competition likely serve to increase trait dissimilarity through the process of limiting similarity. Some communities may also exhibit overdispersion due to external factors or phenological events. For example, the pronounced overdispersion around the Thames estuary may be linked to a contraction of the distribution range of several elasmobranch species into the coastal estuarine areas [86], or because these areas serve as spawning and nursing grounds for some shark and skate species [87]. Both of these mechanisms would lead to an expansion of the existing trait space through unique traits, such as large body size, low fecundity, large offspring size, and high age at maturity; characteristics of elasmobranch species.

Areas of over- or underdispersion potentially reveal not only mechanisms of community assembly, but also information on the potential ecological consequences of biodiversity loss in ecosystems. Whether the loss of an individual species may lead to a functional degradation depend on whether this species carries a unique trait (or combination of traits) or not. In the former case, degradation is likely to occur, especially if the actual trait also supports a particular ecosystem function. In the latter case, functionally similar species (sharing the same traits and ecological niche) may show a compensatory increase, hence buffering for the lost species and ensuring a continued support for any associated ecosystem function. High redundancy within communities may indicate that ecosystem processes and functions are less likely to be altered than in ecosystems exhibiting low redundancy, as each species will account for a disproportionally large amount of the trait diversity in the latter case. Temporal studies of trait redundancy in the North Sea demersal fish communities have shown that trait-wise similar groups with a larger number of species showed higher stability in terms of biomass than groups with fewer species [88]. The degree of trait redundancy in species-rich ecosystems may therefore act as an insurance promoting stability of ecosystem processes and functions against species loss [89–92].

## Conclusions

Protection and conservation efforts are often based on the spatial distribution of biodiversity hotspots, focusing on a single or a few parameters [93]. Differences between biodiversity indicators and trait redundancy are presently receiving increasing attention in the support of management and biodiversity conservation [33,94] as marine and freshwater ecosystems remain vulnerable to loss of species [30,95]. However, trait diversity may still be significantly underrepresented in protected areas [16,96]. This study shows that using trait-based approaches can provide information relevant to conservation and management which could not be obtained through the use of taxonomy-based biodiversity indicators alone. These results emphasize the importance of investigating multiple components of biodiversity (e.g. taxonomy, traits and abundances) to reveal temporal and spatial incongruences, and community assembly rules, but also to inform conservation efforts to protect a broader scope of biodiversity components in general.

## Supporting information

S1 Table Overview of species aggregations into multi-species groups. (DOCX)

S2 Table Abundance to biomass-conversion parameters. (DOCX)

S3 Table Trait information on all species. (DOCX)

S4 Table Summary statistics of natural and anthropogenic environmental covariates used in the relative variable importance analysis. (DOCX)

S1 Fig Relative biomass of all species. (DOCX)

S2 Fig Ratios of TRic to SRic, and TEve to SEve over the study period. (DOCX)

S1 Dataset Biomass of species per year per ICES rectangle. (XLSX)

S2 Dataset Values of biodiversity indicators per year per ICES rectangle. (XLSX)

S3 Dataset Temporally averaged values of natural and anthropogenic environmental covariates. (XLSX)
