# Supplementary material for: Temporal and spatial differences between taxonomic and trait biodiversity in a large marine ecosystem: Causes and consequences
Source: PLoS One. 2017 Dec 18;12(12):e0189731. doi: 10.1371/journal.pone.0189731 (PMC5734758; doi:10.1371/journal.pone.0189731)
Supplement: S1 Table — (DOCX) [file pone.0189731.s001.docx]

| Reported species | Multi-species group |
| --- | --- |
| *Mustelus mustelus*  *Mustelus asterias* | *Mustelus* spp*.* |
| *Callionymus lyra*  *Callionymus maculatus*  *Callionymus reticulates*  *Callionymidae* | *Callionymus* spp. |
| *Aphia minuta*  *Crystallogobius linearis* | Translucent gobies |
| *Liparis liparis*  *Liparis montagui* | *Liparis* spp. |
| *Syngnathus acus*  *Syngnathus rostellatus* | *Syngnathidae* |
| *Ammodytes marinus*  *Ammodytes tobianus*  *Hyperoplus immaculatus*  *Hyperoplus lanceolatus* | *Ammodytidae* |

**S1 Table. Overview of species aggregations into multi-species groups**
