# Supplementary material for: Temporal and spatial differences between taxonomic and trait biodiversity in a large marine ecosystem: Causes and consequences
Source: PLoS One. 2017 Dec 18;12(12):e0189731. doi: 10.1371/journal.pone.0189731 (PMC5734758; doi:10.1371/journal.pone.0189731)
Supplement: S2 Table — (DOCX) [file pone.0189731.s002.docx]

**S2 Table. Abundance to biomass-conversion parameters**

List of all taxa in the study, together with their corresponding length-weight regression parameters *a* and *b*. Length to weight conversion was done using the equation W (weight) = *a*L*^b^*, where L represents the length class (cm) indicated in the DATRAS NS-IBTS data. Parameters were obtained from [1] unless stated otherwise.

| Taxa | *a* | *b* | Reference |
| --- | --- | --- | --- |
| Agonus cataphractus | 0.00910 | 2.9050 |  |
| Amblyraja radiata | 0.00560 | 3.1210 |  |
| Ammodytidae | 0.00473 | 2.9382 | [2,3] |
| Anarhichas lupus | 0.00330 | 3.2491 |  |
| Anguilla anguilla | 0.00060 | 3.3130 |  |
| Argentina silus | 0.00390 | 3.2027 | [2] |
| Argentina sphyraena | 0.00530 | 3.0534 | [2] |
| Arnoglossus laterna | 0.00650 | 3.0960 |  |
| Brosme brosme | 0.00510 | 3.1890 |  |
| Buglossidium luteum | 0.00780 | 3.1280 |  |
| Callionymus spp. | 0.01353 | 2.6857 |  |
| Chelidonichthys cuculus | 0.00450 | 3.2228 | [2] |
| Chelidonichthys lucerna | 0.00800 | 3.0610 |  |
| Ciliata mustela | 0.00640 | 3.0000 |  |
| Ciliata septentrionalis | 0.00550 | 3.1785 |  |
| Cyclopterus lumpus | 0.05870 | 2.9390 |  |
| Dicentrarchus labrax | 0.00740 | 3.0963 | [2] |
| Dipturus batis | 0.00360 | 3.0787 |  |
| Echiichthys vipera | 0.01290 | 2.9470 |  |
| Enchelyopus cimbrius | 0.00350 | 3.1062 |  |
| Entelurus aequoreus | 0.00020 | 3.0000 |  |
| Eutrigla gurnardus | 0.00340 | 3.2600 |  |
| Gadiculus argenteus | 0.01230 | 2.9030 | [4] |
| Gadus morhua | 0.00390 | 3.2434 |  |
| Gaidropsarus vulgaris | 0.01200 | 2.5470 |  |
| Galeorhinus galeus | 0.00980 | 3.0085 |  |
| Gasterosteus aculeatus | 0.00105 | 3.0490 | [5] (freshwater) |
| Glyptocephalus cynoglossus | 0.00130 | 3.4350 |  |
| Helicolenus dactylopterus | 0.15100 | 3.0456 |  |
| Hippoglossoides platessoides | 0.00700 | 2.9780 |  |
| Hippoglossus hippoglossus | 0.23500 | 1.7970 |  |
| Lepidorhombus whiffiagonis | 0.01340 | 2.7460 |  |
| Leucoraja fullonica | 0.00240 | 3.2330 |  |
| Leucoraja naevus | 0.00240 | 3.2330 |  |
| Limanda limanda | 0.00710 | 3.1190 |  |
| Liparis liparis | 0.01220 | 2.9892 |  |
| Liparis montagui | 0.02920 | 2.9490 |  |
| Lophius budegassa | 0.00440 | 3.3450 |  |
| Lophius piscatorius | 0.01660 | 2.9776 |  |
| Lumpenus lampretaeformis | 0.02440 | 2.0439 |  |
| Melanogrammus aeglefinus | 0.00520 | 3.1560 |  |
| Merlangius merlangus | 0.00420 | 3.1842 |  |
| Merluccius merluccius | 0.00360 | 3.1469 |  |
| Microchirus variegatus | 0.00800 | 3.1410 |  |
| Microstomus kitt | 0.00420 | 3.2695 |  |
| Molva molva | 0.00100 | 3.4362 |  |
| Mullus surmuletus | 0.01010 | 3.0201 |  |
| Mustelus spp. | 0.00410 | 2.9185 |  |
| Petromyzon marinus | 0.00080 | 3.1956 |  |
| Pholis gunnellus | 0.00430 | 3.0180 |  |
| Phrynorhombus norvegicus | 0.00780 | 3.1457 |  |
| Platichthys flesus | 0.00870 | 3.0978 |  |
| Pleuronectes platessa | 0.00780 | 3.0541 |  |
| Pollachius pollachius | 0.00610 | 3.1150 |  |
| Pollachius virens | 0.00420 | 3.1753 |  |
| Pomatoschistus minutus | 0.00620 | 3.1730 |  |
| Raja brachyura | 0.00280 | 3.2330 |  |
| Raja clavata | 0.00320 | 3.1940 |  |
| Raja montagui | 0.00230 | 3.2051 |  |
| Scophthalmus maximus | 0.00460 | 3.3972 |  |
| Scophthalmus rhombus | 0.00550 | 3.3047 |  |
| Scyliorhinus canicula | 0.00310 | 3.0290 |  |
| Sebastes viviparus | 0.01150 | 3.1369 |  |
| Solea solea | 0.00380 | 3.2751 |  |
| Spinachia spinachia | 0.00210 | 3.0000 | [3] |
| Squalus acanthias | 0.00340 | 3.0955 |  |
| Syngnathus spp. | 0.00010 | 3.4705 |  |
| Trachinus draco | 0.00180 | 3.4099 |  |
| Translucent gobies | 0.00464 | 3.2407 |  |
| Triglops murrayi | 0.00880 | 3.0000 |  |
| Trisopterus esmarkii | 0.00460 | 3.1405 |  |
| Trisopterus luscus | 0.00380 | 3.3665 |  |
| Trisopterus minutus | 0.00920 | 3.0265 |  |
| Zeugopterus punctatus | 0.01390 | 3.1457 |  |
| Zeus faber | 0.02290 | 2.9343 | [2] |
| Zoarces viviparus | 0.04170 | 2.2532 |  |

1. Fung T, Farnsworth KD, Reid DG, Rossberg AG. Recent data suggest no further recovery in North Sea Large Fish Indicator. ICES J Mar Sci. 2012;69(2):235–9.

2. Coull KA, Jermyn AS, Newton AW, Henderson GI, Hall WB. Length/Weight Relationships for 88 Species of Fish Encountered in the North East Atlantic. Scottish Fisheries Research Report. Aberdeen; 1989.

3. Froese R, Pauly D. Fishbase [Internet]. World Wide Web electronic publication. 2015. Available from: www.fishbase.org

4. Robinson LA, Greenstreet SPR, Reiss H, Callaway R, Craeymeersch J, De Boois I, et al. Length–weight relationships of 216 North Sea benthic invertebrates and fish. J Mar Biol Assoc United Kingdom. 2010;90(1):95–8.

5. Verreycken H, Van Thuyne G, Belpaire C. Length-weight relationships of 40 freshwater fish species from two decades of monitoring in Flanders (Belgium). J Appl Ichthyol. 2011;27(6):1416–21.
