# Supplementary material for: Temporal and spatial differences between taxonomic and trait biodiversity in a large marine ecosystem: Causes and consequences
Source: PLoS One. 2017 Dec 18;12(12):e0189731. doi: 10.1371/journal.pone.0189731 (PMC5734758; doi:10.1371/journal.pone.0189731)
Supplement: S3 Table — (DOCX) [file pone.0189731.s003.docx]

**S3 Table. Trait information on all taxa**

List of taxa with trait information and references. (L∞ (cm) (von Bertalanffy length at infinity); Age at 50% maturity (years) (age at which 50% of the population become mature for the first time); Fecundity (Average number of eggs per adult female for one spawning season); Offspring size (cm) (Size of the released eggs or young (in case of live birth)); Body shape (shape of body); Diet (main dietary group(s)); Spawning behavior (reproductive mode); Fin shape (the shape of the caudal fin). * inferred from *A. silus*, ** inferred from *L. naevus*, *** inferred from *P. pollachius*

| **Taxa** | **Common name** | **L_∞_** | **Age 50% maturity** | **Fecundity** | **Offspring size** | **Body shape** | **Diet** | **Spawning**  **strategy** | **Fin shape** | **Reference** |
| --- | --- | --- | --- | --- | --- | --- | --- | --- | --- | --- |
| *Agonus cataphractus* | Armed bullhead | 15 | 1.0 | 3000 | 2 | Normal | Benthivore | Ov | Truncated | [1–5] |
| *Amblyraja radiata* | Starry ray | 68 | 3.5 | 17 | 110 | Flat | Piscivore | Ob | Continuous | [1–3,5] |
| *Ammodytidae* |  | 33 | 2.4 | 14613 | 0.8 | Elongated | Plankto-piscivore | Ob | Forked | [3,5] |
| *Anarhichas lupus* | Wolffish | 113 | 6.5 | 18870 | 5 | Elongated | Benthivore | Og | Truncated | [1–3,5,6] |
| *Anguilla Anguilla* | European eel | 72 | 11.0 | 2500000 | 1 | Eel-like | Bentho-piscivore | Op | Continuous | [2,3,5,7,8] |
| *Argentina silus* | Greater silver smelt | 41 | 5.6 | 13500 | 3.25 | Elongated | Planktivore | Op | Forked | [2,5]5,2 |
| *Argentina sphyraena* | Lesser seilver smelt | 22 | 2.7 | 13500* | 1.8 | Elongated | Planktivore | Op | Forked | [2,5] |
| *Arnoglossus laterna* | Scaldfish | 15 | 8.0 | 33333 | 0.8 | Normal | Benthivore | Op | Truncated | [1,5,9] |
| *Brosme brosme* | Tusk | 75 | 7.0 | 2013500 | 1.4 | Elongated | Benthivore | Op | Rounded | [5,6,10,11] |
| *Buglossidium luteum* | Solenette | 11 | 3.0 | 13400 | 0.8 | Flat | Benthivore | Op | Rounded | [1,5,12,13] |
| *Callionymus spp.* |  | 18 | 2.5 | 3500 | 0.8 | Normal | Benthivore | Op | Rounded | [5,12,14] |
| *Chelidonichthys cuculus* |  | 39 | 3.7 | 100000 | 1.3 | Normal | Benthivore | Op | Truncated | [2,5,15] |
| *Chelidonichthys lucerna* |  | 57 | 3.5 | 100000 | 13 | Normal | Benthivore | Op | Truncated | [2,3,5] |
| *Ciliata mustela* | Five-bearded rockling | 25 | 1.0 | 19500 | 0.8 | Elongated | Benthivore | Op | Rounded | [2,3,5,16] |
| *Ciliata septentrionalis* | Northern rockling | 16 | 1.0 | 19500 | 0.8 | Elongated | Benthivore | Op | Rounded | [2,3,5,17] |
| *Cyclopterus lumpus* | Lumpsucker | 53 | 3.5 | 194112 | 2.3 | Short/deep | Bentho-piscivore | Og | Truncated | [2,3,5,18] |
| *Dicentrarchus labrax* | European seabass | 79 | 5.5 | 520278 | 1.3 | Normal | Piscivore | Op | Forked | [2,3,5,19,20] |
| *Dipturus batis* | Common skate | 254 | 11.0 | 40 | 170 | Flat | Piscivore | Ob | Continuous | [5,21,22] |
| *Echiichthys vipera* | Lesser weever fish | 15 | 1.0 | 57600 | 1.2 | Normal | Bentho-piscivore | Og | Truncated | [2,5,23] |
| *Enchelyopus cimbrius* | Four-bearded rockling | 36 | 3.0 | 25000 | 0.9 | Elongated | Benthivore | Op | Rounded | [5,6] |
| *Entelurus aequoreus* | Snake pipefish | 47 | 2.0 | 700 | 1 | Eel-like | Planktivore | Os | Truncated | [5,17,24] |
| *Eutrigla gurnardus* | Grey gurnard | 36 | 3.0 | 250000 | 1.4 | Normal | Benthivore | Op | Emarginate | [1–3,5,12] |
| *Gadus morhua* | Cod | 115 | 3.8 | 1000000 | 1.43 | Normal | Piscivore | Op | Truncated | [2,3,5,12] |
|  |  |  |  |  |  |  |  |  |  |  |
|  |  |  |  |  |  |  |  |  |  |  |
| **Taxa** | **Common name** | **L_∞_** | **Age 50% maturity** | **Fecundity** | **Offspring size** | **Body shape** | **Diet** | **Spawning**  **strategy** | **Fin shape** | **Reference** |
| *Gaidropsarus vulgaris* | Three-bearded rockling | 48 | 3.0 | 11018375 | 0.8 | Elongated | Benthivore | Op | Rounded | [3,5,23,25] |
| *Galeorhinus galeus* | Tope shark | 158 | 9.5 | 29 | 240 | Elongated | Piscivore | V | Heterocercal | [2,4,5,26] |
| *Gasterosteus aculeatus* | Three-spinned stickleback | 9 | 1.0 | 250 | 1.3 | Normal | Benthivore | Og | Truncated | [2,3,5,27] |
| *Glyptocephalus cynoglossus* | Witch | 44 | 4.5 | 278550 | 1.27 | Flat | Benthivore | Op | Truncated | [2,5,6,12] |
| *Helicolenus dactylopterus* | Bluemouth redfish | 34 | 14.3 | 230055 | 2.8 | Normal | Bentho-piscivore | V | Truncated | [2,5,28] |
| *Hippoglossoides platessoides* | Long rough dab | 25 | 2.6 | 1525000 | 2.5 | Flat | Bentho-piscivore | Op | Rounded | [1,5,12] |
| *Hippoglossus hippoglossus* | Halibut | 204 | 6.7 | 1900000 | 3.6 | Flat | Piscivore | Op | Truncated | [1,5,25] |
| *Lepidorhombus whiffiagonis* | Megrim | 52 | 2.8 | 333523 | 0.92 | Flat | Piscivore | Op | Rounded | [2,5,29,30] |
| *Leucoraja fullonica* | Shagreen ray | 123 | 7.0** | 90** | 60** | Flat | Piscivore | Ob | Continuous | [5,17,23] |
| *Leucoraja naevus* | Cuckoo ray | 71 | 7.0 | 90 | 60 | Flat | Piscivore | Ob | Continuous | [2,5,21] |
| *Limanda limanda* | Dab | 27 | 2.3 | 100000 | 1.2 | Flat | Benthivore | Op | Truncated | [1,5,31] |
| *Liparis liparis* | Sea snail | 12 | 0.9 | 460 | 1.5 | Elongated | Benthivore | Ov | Truncated | [2,5,17,23] |
| *Lophius budegassa* | Black-bellied anglerfish | 84 | 7.7 | 1550000 | 1.8 | Short/deep | Piscivore | Op | Truncated | [2,5,32] |
| *Lophius piscatorius* | Anglerfish | 106 | 4.5 | 1000000 | 2.7 | Short/deep | Piscivore | Op | Truncated | [1,5,7,33] |
| *Lumpenus lampretaeformis* | Snake blenny | 48 | 3.0 | 1000 | 11 | Eel-like | Benthivore | Og | Rounded | [1,5,17] |
| *Melanogrammus aeglefinus* | Haddock | 68 | 2.5 | 535000 | 1.5 | Normal | Benthivore | Op | Truncated | [5,12,25] |
| *Merlangius merlangus* | Whiting | 43 | 1.5 | 350800 | 1.28 | Normal | Piscivore | Ob | Truncated | [5,7,12] |
| *Merluccius merluccius* | European hake | 105 | 6.8 | 294521 | 1 | Elongated | Piscivore | Op | Truncated | [1,34,35] |
| *Microchirus variegatus* | Thickback sole | 19 | 3.0 | 500000 | 1.3 | Flat | Benthivore | Op | Rounded | [2,5,23] |
| *Microstomus kitt* | Lemon sole | 36 | 4.0 | 28988 | 1.2 | Flat | Benthivore | Op | Rounded | [2,5,12] |
| *Molva molva* | Common ling | 183 | 6.5 | 40000000 | 1 | Elongated | Piscivore | Op | Rounded | [1,5,12,36] |
| *Mullus surmuletus* | Striped red mullet | 40 | 1.5 | 10000 | 0.85 | Normal | Benthivore | Op | Forked | [2,3,5,23] |
| *Mustelus spp.* | Smoothhounds | 134 | 5.0 | 16 | 350 | Elongated | Benthivore | V | Heterocercal | [1,2,5] |
| *Petromyzon marinus* | Sea lamprey | 90 | 7.0 | 228000 | 0.9 | Eel-like | Piscivore | Og | Continuous | [5,17] |
| *Pholis gunnellus* | Gunnel | 26 | 2.0 | 100 | 1.6 | Eel-like | Benthivore | Og | Rounded | [2,3,5] |
| *Phrynorhombus norvegicus* | Norwegian topknot | 13 | 1.3 | 2666761 | 0.82 | Flat | Bentho-piscivore | Op | Rounded | [1,5,37] |
| *Platichthys flesus* | Flounder | 33 | 3.5 | 650000 | 1.06 | Flat | Benthivore | Op | Rounded | [2,3,23] |
| *Pleuronectes platessa* | European plaice | 61 | 2.5 | 146778 | 1.8 | Flat | Benthivore | Op | Rounded | [2,3,25] |
| *Pollachius pollachius* | Pollack | 86 | 2.5 | 220000 | 1.1 | Normal | Piscivore | Op | Emarginate | [2,3,5,23] |
| *Pollachius virens* | Saithe | 177 | 4.6 | 4831000 | 1.1 | Normal | Piscivore | Op*** | Emarginate | [1,12,38] |
| *Pomatoschistus minutus* | Sand goby | 9 | 0.9 | 3654 | 0.8 | Normal | Benthivore | Og | Rounded | [5,23] |
| *Raja clavata* | Thornback ray | 108 | 7.0 | 61 | 65 | Flat | Bentho-piscivore | Ov | Continuous | [2,5,39] |
| *Raja montagui* | Spotted ray | 77 | 5.0 | 43 | 60 | Flat | Bentho-piscivore | Ov | Continuous | [2,5,39] |
|  |  |  |  |  |  |  |  |  |  |  |
| **Taxa** | **Common name** | **L_∞_** | **Age 50% maturity** | **Fecundity** | **Offspring size** | **Body shape** | **Diet** | **Spawning**  **strategy** | **Fin shape** | **Reference** |
| *Scophthalmus maximus* |  | 52 | 1.5 | 5000000 | 1 | Flat | Piscivore | Op | Rounded | [2,5,23] |
| *Scophthalmus rhombus* | Brill | 51 | 3.0 | 5000000 | 1.3 | Flat | Piscivore | Op | Rounded | [2,5] |
| *Scyliorhinus canicula* | Lesser spotted dogfish | 81 | 5.0 | 46 | 40 | Elongated | Bentho-piscivore | Ov | Heterocercal | [2,5,6,40] |
| *Sebastes viviparous* | Norway haddock | 27 | 20.0 | 8558 | 5.5 | Normal | Piscivore | V | Truncated | [2,5,6] |
| *Solea solea* | Sole | 35 | 4.0 | 118050 | 1.2 | Flat | Benthivore | Op | Rounded | [2,5,12] |
| *Spinachia spinachia* | Sea stickleback | 17 | 1.0 | 175 | 2 | Elongated | Benthivore | Og | Truncated | [1,2,5] |
| *Squalus acanthias* | Spurdog | 90 | 6.5 | 11 | 150 | Elongated | Piscivore | V | Heterocercal | [1,2,6,41] |
| *Syngnathus spp.* | Pipefish | 33 | 1.0 | 186 | 1.8 | Eel-like | Planktivore | Os | Rounded | [1,2,5] |
| *Trachinus draco* | Greater weever fish | 42 | 1.0 | 38926 | 1 | Normal | Bentho-piscivore | Op | Truncated | [17,42,43] |
| *Translucent gobies* |  | 12 | 0.5 | 3577 | 0.98 | Normal | Planktivore | Og | Truncated | [1,2,5] |
| *Triglops murrayi* | Sculpin | 16 | 3.5 | 100 | 1.75 | Elongated | Benthivore | Og | Truncated | [3,17,36] |
| *Trisopterus esmarkii* | Norway pout | 21 | 3.5 | 220000 | 1.2 | Normal | Bentho-piscivore | Op | Truncated | [2,5,6] |
| *Trisopterus luscus* | Bib | 35 | 2.0 | 205595 | 1.1 | Normal | Benthivore | Op | Truncated | [2,5,12,23] |
| *Trisopterus minutus* | Poor cod | 20 | 2.0 | 10000 | 1 | Normal | Benthivore | Op | Emarginate | [1,2,5,6] |
| *Zeugopterus punctatus* | Topknot | 20 | 1.4 | 2666761 | 1 | Flat | Bentho-piscivore | Op | Rounded | [17,37,44] |
| *Zeus faber* | John dory | 59 | 3.5 | 292500 | 2 | Short/deep | Piscivore | Op | Truncated | [1,2,5] |
| *Zoarces viviparus* | Norway haddoc | 30 | 1.5 | 70 | 3 | Eel-like | Benthivore | V | Continuous | [1,2,5] |

1. Muus BJ, Nielsen JG. Sea fish: Scandinavian Fishing Year Book. Hedehusene; 1999. 340 p.

2. Heessen HJL, Daan N, Ellis JR. Fish atlas of the Celtic Sea, North Sea, and Baltic Sea. Heessen HJL, Daan N, Ellis JR, editors. Wageningen: Wageningen Academic Publishers; 2015. 572 p.

3. Franco A, Elliott M, Franzoi P, Torricelli P. Life strategies of fishes in European estuaries: The functional guild approach. Mar Ecol Prog Ser. 2008;354:219–28.

4. Walker PA, Hislop JRG. Sensitive skates or resilient rays? Spatial and temporal shifts in ray species composition in the central and north-western North Sea between 1930 and the present day. ICES J Mar Sci. 1998;55:392–402.

5. Froese R, Pauly D. Fishbase [Internet]. World Wide Web electronic publication. 2015. Available from: www.fishbase.org

6. Jennings S, Alvsvåg J, Cotter AJR, Ehrich S, Greenstreet SPR, Jarre-Teichmann A, et al. Fishing effects in northeast atlantic shelf seas: Patterns in fishing effort, diversity and community structure. VI. Gale effects on vertical distribution and structure of a fish assemblage in the North Sea. Fish Res. 1999;40(2):125–34.

7. Denney NH, Jennings S, Reynolds JD. Life-history correlates of maximum population growth rates in marine fishes. Proc Biol Sci. 2002;269(1506):2229–37.

8. Narberhaus I, Krause J, Bernitt U. Threatened biodiversity in the German North and Baltic seas - Naturschutz und Biologische Vielfalt, Heft 117. Bonn: Federal Agency for Nature Conservation; 2012.

9. Gibson RN, Ezzi IA. The biology of the scaldfish, Arnoglossus laterna (Walbaum) on the west coast of Scotland. J Fish Biol. 1980;(January 1975):575.

10. W.S. Oldham. Biology of the Scotian Shelf cusk, Brosme brosme. ICNAF Res Bull. 1972;9:85–98.

11. Tyus HM. Ecology and Conservation of Fishes. CRC Press. 2012;

12. Perry AL, Low PJ, Ellis JR, Reynolds JD. Climate Change and Distribution Shifts in Marine Fishes. Science (80- ). 2005;308(5730):1912–5.

13. Van der Land MA. Distribution of flatfish eggs in the 1989 egg surveys in the southeastern North Sea, and mortality of plaice and sole eggs. Netherlands J Sea Res [Internet]. 1991 Jul [cited 2017 Feb 13];27(3–4):277–86. Available from: http://linkinghub.elsevier.com/retrieve/pii/0077757991900305

14. Van Der Veer HW, Creutzberg F, Dapper R, Duineveld GCA, Fonds M, Kuipers BR, et al. On the ecology of the dragonet Callionymus lyra L. in the southern North Sea. Netherlands J Sea Res. 1990;26(15):139–50.

15. Marriott AL, Latchford JW, McCarthy ID. Population biology of the red gurnard (Aspitrigla cuculus L.; Triglidae) in the inshore waters of Eastern Anglesey and Northwest Wales. J Appl Ichthyol. 2010;26(4):504–12.

16. Cohen DM, Lnada T, Lwamoto T, Scialabba N. An Annotated and Illustrated Catalogue of Cods, Hakes, Grenadiers and other Gadiform Fishes Known to Date. Vol.10 Gad. Vol. 7, FAO species catalogue - Vol.10. Gadiform Fishes of the world (Order Gadiformes). Rome: Food and Agriculture Organization of the United Nations; 1990. 442 p.

17. Greenstreet SPR, Rossberg AG, Fox CJ, Quesne WJF Le, Blasdale T, Boulcott P, et al. Demersal fish biodiversity: species-level indicators and trends-based targets for the Marine Strategy Framework Directive. 2012;69:1789–801.

18. Christiansen JS, Fevolden SE, Karamushko OV, Karamushlo LI. Reproductive traits of marine fish in relation to their mode of oviposition and zoogeographic distribution. In: ICES CM 19. 1997. p. 14.

19. Cambiè G, Kaiser MJ, Hiddink JG, Salomonsen H, Pantin JR, Mccarthy I. Population dynamics of the European sea bass (Dicentrarchus labrax) in Welsh waters and management implications. 2015;(56):76.

20. Wassef E, El Emary H. Contribution to the biology of bass, Dicentrarchus labrax L. in the Egyptian Mediterranean waters off Alexandria. Cymbrium. 1989;13(4):327–45.

21. Du Buit MH. Age et croissance de Raja batis et de Raja naevus en Mer Celtique. ICES J Mar Sci. 1972;37(3):261–5.

22. Neal, K., Pizzolla, P. & Wilding C 2008. Dipturus batis. Common Skate. Marine Life Information Network: Biology and Sensitivity Key Information Sub-programme [online]. 2008.

23. Pecuchet L, Lindegren M, Hidalgo M, Delgado M, Esteban A, Fock HO, et al. Reproductive traits (Fecundity, egg diameter, parental care) of marine European fish. 2016.

24. Braga Goncalves I, Ahnesjö I, Kvarnemo C. The relationship between female body size and egg size in pipefishes. J Fish Biol. 2011;78(6):1847–54.

25. Jennings S, Reynolds JD, Mills SC. Life history correlates of responses to Fisheries exploitation. Proc R Soc London B. 1998;265(November 1997):333–9.

26. Walker TI, Cavanagh RD, Stevens JD, Carlisle AB, Chiaramonte GE, Domingo A, et al. Galeorhinus galeus. The IUCN Red List of Threatened Species. 2006.

27. Maitland PS, Campbell RN. Freshwater fishes of the British Isles. London: HarperCollins Publishers; 1992. 368 p.

28. Pécuchet L, Törnroos A, Lindegren M. Patterns and drivers of fish community assembly in a large marine ecosystem. Mar Ecol Prog Ser. 2016;546:239–48.

29. Robson S. Age, growth, reproductive biology and population dynamics of the common megrim Lepidorhombus whiffiagonis (Walbaum, 1792) from off the west coast of Ireland). Galway-Mayo Institute of Technology; 2004.

30. Acom ICM. ICES IBP MEGRIM REPORT 2016 ( West and Southwest of Ireland , Bay of Biscay ) in Divisions 7 . b – k and 8 . a , 8 . b , and 8 . d ( IBP Megrim 2016 ) Megrim ( Lepidorhombus whiffiagonis ) Inter-Benchmark Protocol Workshop International Council for the Ex. 2016;(July 2015).

31. Rijnsdorp AD, Vethaak AD, Van Leeuwen PI. Population biology of dab Limanda limanda in the southeastern North Sea. Mar Ecol Prog Ser. 1992;91(1–3):19–35.

32. Armstrong MP, Musick JA, Colvocoresses JA. Age, growth, and reproduction of the goosefish Lophius americanus(Pisces: Lophiiformes). Fish Bull. 1992;90(2):217–30.

33. Afonso-Dias IP, Hislop JRG. The reproduction of anglerfish Lophius piscatorius Linnaeus from the north-west coast of Scotland. J Fish Biol. 1996 Dec;49(sa):18–39.

34. Belloc G. Etude Monographique du Merlu (Merlucius merluccius L.). Rev Trav Off Pêches marit. 1929;2:153–99.

35. Mehault S, Domínguez-Petit R, Cerviño S, Saborido-Rey F. Variability in total egg production and implications for management of the southern stock of European hake. Fish Res. 2010;104(1–3):111–22.

36. Wienerroither R, Johannesen E, Dolgov A, Byrkjedal I, Bjelland O, Drevetnyak K, et al. Atlas of the Barents Sea fishes. IMR/PINRO Joint Report Series 1-2011. 2011.

37. ICES. Report of the Planning Group on North Sea Cod and Plaice Egg Surveys (PGEGGS). 2009;2–3 Decemb. Available from: http://www.ices.dk/sites/pub/Publication Reports/Expert Group Report/SSGESST/2009/PGEGGS/PGEGGS09.pdf

38. Storozhuk AY, Goloyanov AV, Golubyatnikova IP. On the fecundity of Saithe (Pollachius virens L.) in the North Sea. In: ICES: CM F(13) 4. 1974.

39. Gallagher MJ, Nolan CP, Jeal F. Age, growth and maturity of the commercial ray species from the Irish Sea. J Northwest Atl Fish Sci. 2005;35(November 2004):47–66.

40. Ellis JR, Shackley SE. The reproductive biology of Scyliorhinus canicula in the Bristol Channel, U.K. J Fish Biol [Internet]. 1997;51(2):361–72. Available from: http://dx.doi.org/10.1111/j.1095-8649.1997.tb01672.x

41. Stenberg C. Life history of the piked dogfish (Squalus acanthias L.) in Swedish waters. J Northwest Atl Fish Sci. 2005;35(March):155–64.

42. Ak O, Genç Y. Growth and reproduction of the greater weever (Trachinus draco L ., 1758) along the eastern coast of the Black Sea. J Black Sea/ Mediterr Environ. 2013;19(1):95–110.

43. Bagge O. The biology of the greater weever (Trachinus draco) in the commercial fishery of the Kattegat. ICES J Mar Sci. 2004;61(6):933–43.

44. P.J.P Whitehead, M-L Bauchot, J-C Hureau, J Nielsen ET. Fishes of the north-eastern Atlantic and the Mediterranean, volume 3. United Nations Educational Scientific and Cultural Organization; 1986.
