# Supplementary material for: Temporal and spatial differences between taxonomic and trait biodiversity in a large marine ecosystem: Causes and consequences
Source: PLoS One. 2017 Dec 18;12(12):e0189731. doi: 10.1371/journal.pone.0189731 (PMC5734758; doi:10.1371/journal.pone.0189731)
Supplement: S4 Table — (DOCX) [file pone.0189731.s004.docx]

**S4 Table. Summary statistics of natural and anthropogenic environmental covariates used in the Relative Variable Importance analysis**

| **Covariate** | **Minimum** | **Maximum** | **Mean** | **Median** | **1^st^ quartile** | **3^rd^ quartile** |
| --- | --- | --- | --- | --- | --- | --- |
| **Depth (m) *** | 23.39 | 162.22 | 76.83 | 73.50 | 45.08 | 104.79 |
| **Sea bottom temperature (°C) **** | 5.712 | 8.761 | 6.912 | 6.856 | 6.352 | 7.395 |
| **Sea bottom Salinity **** | 34.07 | 35.33 | 34.93 | 34.93 | 34.74 | 35.15 |
| **Temperature seasonality (Δ°C) **** | - 0.270 | 10.181 | 3.172 | 1.779 | 0.640 | 5.108 |
| **Salinity variability **** | 0.0112 | 0.4953 | 0.0738 | 0.0564 | 0.0309 | 0.0835 |
| **PCI ***** | 0.4530 | 2.0680 | 1.0669 | 1.1530 | 0.5795 | 1.4325 |
| **Substrate richness ****** | 1 | 6 | 2.697 | 3 | 2 | 3 |
| **Substrate evenness ****** | 0.3518 | 1.0000 | 0.7475 | 0.7474 | 0.5971 | 0.9320 |
| **Beam effort (h) ******* | 0.84 | 39642.37 | 4965.34 | 1770.44 | 88.27 | 5580.16 |
| **Otter effort (h) ******* | 717.5 | 66489.0 | 6345.2 | 3568.6 | 1930.8 | 7260.4 |

Sources and dates downloaded: *<http://datras.ices.dk/Home/Default.aspx>; December 2015, **[1]; May 2016, *** <https://www.sahfos.ac.uk/>; See Data Availability Statement, May 2016, ****<http://www.emodnet.eu/seabed-habitats>; May 2016, ***** See Data Availability Statement

1. Núñez-Riboni I, Akimova A. Monthly maps of optimally interpolated in situ hydrography in the North Sea from 1948 to 2013. J Mar Syst. 2015;151:15–34.
