# Supplementary material for: Temporal and spatial differences between taxonomic and trait biodiversity in a large marine ecosystem: Causes and consequences
Source: PLoS One. 2017 Dec 18;12(12):e0189731. doi: 10.1371/journal.pone.0189731 (PMC5734758; doi:10.1371/journal.pone.0189731)
Supplement: S1 Fig — (DOCX) [file pone.0189731.s005.docx]

**S1 Fig. Barplot of relative biomass of all taxa.**
