# Supplementary material for: Temporal and spatial differences between taxonomic and trait biodiversity in a large marine ecosystem: Causes and consequences
Source: PLoS One. 2017 Dec 18;12(12):e0189731. doi: 10.1371/journal.pone.0189731 (PMC5734758; doi:10.1371/journal.pone.0189731)
Supplement: S2 Fig — (DOCX) [file pone.0189731.s006.docx]

**S2 Fig. Ratios of TRic to SRic and TEve to SEve over the study period**


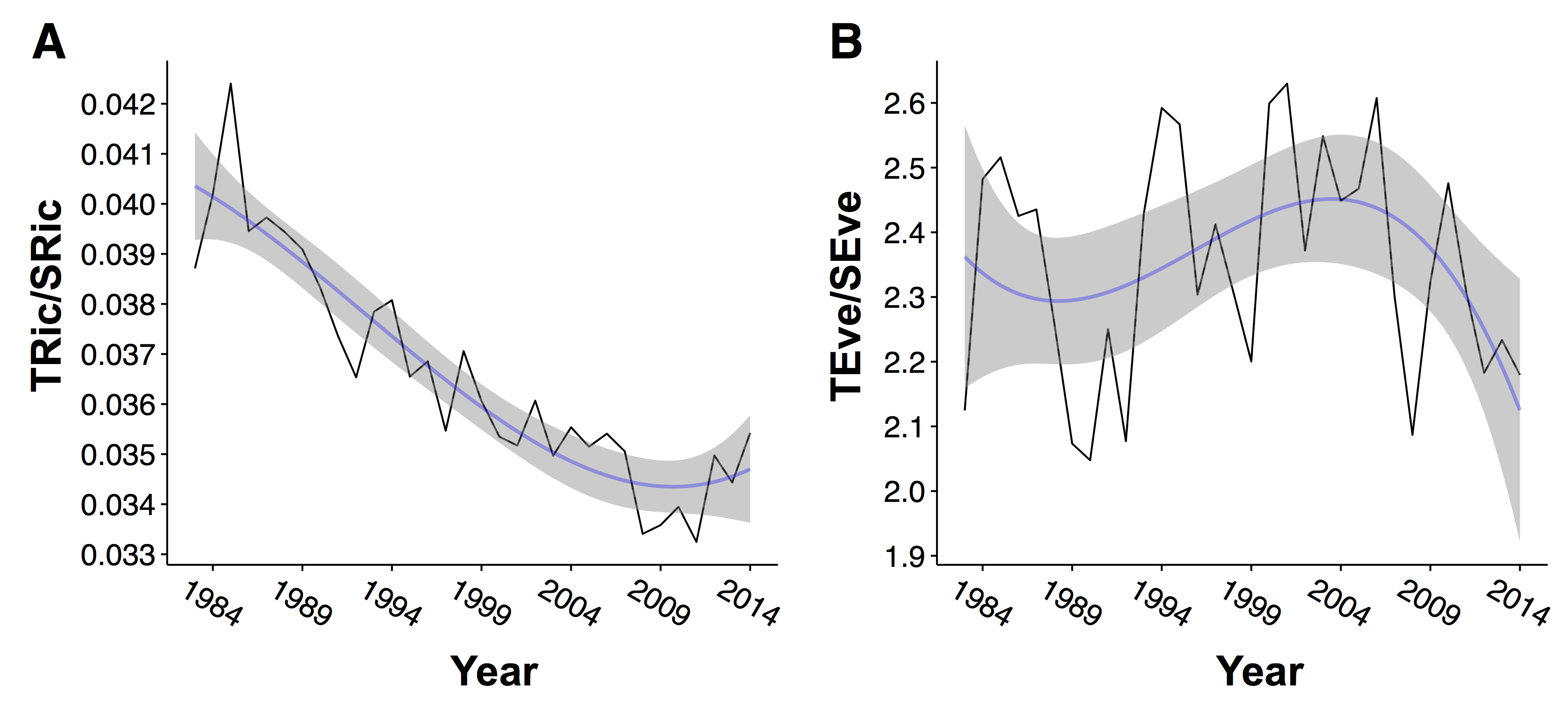


Temporal trends of ratios between TRic and SRic (A), and TEve and SEve (B). Blue curves represent fitted Generalized Additive Models. Grey shaded areas represent 95% confident intervals.
